# Collaborative care compared to enhanced standard treatment of depression with co-morbid medical conditions among patients from rural South India: a cluster randomized controlled trial (HOPE Study)

**DOI:** 10.1186/s12888-022-04000-3

**Published:** 2022-06-13

**Authors:** Krishnamachari Srinivasan, Elsa Heylen, R. Johnson Pradeep, Prem K. Mony, Maria L. Ekstrand

**Affiliations:** 1 Division of Mental Health & Neurosciences, St John’s Research Institute, Bangalore, India; 2 Department of Psychiatry, St John’s Medical College, Bangalore, India; 3 Division of Prevention Sciences, University of California, San Francisco, USA; 4 Division of Epidemiology and Community Health, St John’s Medical College & Research Institute, Bangalore, India

**Keywords:** Collaborative care model, Depression, Chronic medical conditions, Primary health center, Community health workers

## Abstract

**Background:**

Depression is common among primary care patients in LMIC but treatments are largely ineffective. In this cluster-randomized controlled trial, we tested whether depression outcomes are different among recipients of a collaborative care model compared to enhanced standard treatment in patients with co-morbid chronic medical conditions.

**Methods:**

We conducted a cluster randomized controlled trial among participants 30 years or older seeking care at 49 primary health centers (PHCs) in rural Karnataka, diagnosed with major depressive disorder, dysthymia, generalized anxiety disorder, or panic disorder on the MINI-International Neuropsychiatric Interview plus either hypertension, diabetes, or ischemic heart disease. From a list of all PHCs in the district, 24 PHCs were randomized a priori to deliver collaborative care and 25 PHCs enhanced standard treatment. The collaborative care model consisted of a clinic-based and a community-based component. Study assessment staff was blinded to treatment arm allocation. The primary outcome was the individual-level PHQ-9 score over time.

**Results:**

Between May 2015 and Nov 2018, 2486 participants were enrolled, 1264 in the control arm, and 1222 in the intervention arm. They were assessed at baseline, 3, 6 and 12 months. The mean PHQ-9 depression score was around 8.5 at baseline. At each follow-up PHQ-9 scores were significantly lower in the intervention (5.24, 4.81 and 4.22 at respective follow-ups) than in the control group (6.69, 6.13, 5.23, respectively). A significant time-by-treatment interaction (*p* < 0.001) in a multi-level model over all waves, nested within individuals who were nested within PHCs, confirmed that the decrease in depression score from baseline was larger for collaborative care than enhanced standard care throughout follow-up.

**Conclusions:**

The collaborative care intervention resulted in significantly lower depression scores compared to enhanced standard care among participants with co-morbid physical conditions. The findings have potential implications for integrating mental health and chronic disease treatment in resource constrained settings.

**Trial registration:**

ClinicalTrials.govNCT02310932, registered on December 8, 2014, and Clinical Trials Registry India CTRI/2018/04/013001, registered on April 4, 2018. Retrospectively registered.

**Supplementary Information:**

The online version contains supplementary material available at 10.1186/s12888-022-04000-3.

## Introduction

In India, it is estimated that 30–34% of primary care patients have a primary diagnosis of depression and/or anxiety disorders, often referred to as common mental disorders (CMD) [1]. Several of these patients are recipients of ineffective symptomatic treatment [2]. While depression can be effectively treated in primary health care (PHC) settings, only 10–25% of these patients seek treatment due to lack of awareness and/or perceived stigma [3, 4].

Depression, independent of other risk factors, increases the risk for developing cardiovascular diseases (CVD) and adversely impacts cardiac outcomes [5–7]. In addition, the presence of chronic medical conditions increases the risk for depressive disorders [8]. Several coronary risk factors such as hypertension, diabetes mellitus (DM), insulin resistance, and dyslipidemia are highly prevalent in rural India [9]. The Non-Communicable Disease Risk Factor Collaboration study reported a significant increase in age-standardized diabetes and hypertension prevalence among both men and women in India in the period from 1980 to 2014 [10]. Thus, rising rates of chronic medical conditions provide indirect evidence of an increase in psychiatric prevalence due to co-morbid anxiety and depression [11].

Most non-communicable diseases share common risk factors, making them candidates for integrated intervention approaches. A US-based study noted that integrating treatment of depression with DM and/or CVD resulted in a greater overall improvement in glycosylated haemoglobin, LDL cholesterol, systolic blood pressure, and depressive symptoms [12]. In addition, treatment of depression among patients with CVD resulted in a significantly lower risk of secondary cardiovascular events [13]. Integrating treatment of depression with CVD and/or DM will optimally use resources resulting in greater cost-effectiveness as both conditions have multiple encounters with health systems and cross benefit from common behavioural strategies [14, 15].

There is a scarcity of trained health care providers in India [16] with less than one psychiatrist per million citizens in rural India [17]. The availability of psychologists, social workers, and psychiatry nurses is even smaller, pointing to the need for training primary health care staff to close the treatment gap [18].

The Collaborative Care Model (CCM) in which care managers and consultant psychiatrists support PHC-based health providers, improved depression outcomes in US primary care settings [19], is cost-effective [20], and sustainable [21]. A systematic review of 79 RCTs with 24,308 participants showed that both short-term and long-term outcomes for depression improved significantly among recipients in the collaborative care arm [22]. However, some trials had only a primary care provider and care manager as part of the collaborative care team without the support of a mental health specialist. In addition, most studies did not report if the trials included participants with co-morbid physical conditions [23]. The review also noted that there is a limited evidence base for the efficacy of CCM for depression in low- and middle-income countries (LMIC). While a previous study from India showed that a lay health worker led intervention using stepped collaborative care improved depression outcomes in a PHC setting [24], our study extends the integrated CCM to patients diagnosed with both depression and chronic medical conditions.

In this cluster randomized controlled trial, we aimed to compare CCM to enhanced standard treatment in improving depression among participants from rural south India who were diagnosed with co-morbid chronic medical conditions. Cluster randomization (unit: PHCs) was necessary because the intervention requires a clinic- and community-based approach, so it would have been impossible to provide both kinds of treatment in one PHC, given its limited staff and also the fact that virtually all inhabitants of its catchment area visit this one PHC for their primary health care needs.

## Methods

### Study design and participants

The HOPE study (Healthier Options through Empowerment) was a cluster randomized controlled trial to evaluate the effects of a collaborative care intervention compared to enhanced standard treatment on the diagnosis and treatment of depression in patients with co-morbid diabetes and/or cardiovascular conditions. The trial took place in 49 primary health centres (the clusters) in the rural Ramanagaram district of Karnataka state in southern India [25]. All PHCs in the district were eligible for initial inclusion (see randomization below). Eligible participants were PHC patients 30 years or older, with a diagnosis of major depressive disorder, dysthymia, generalized anxiety disorder, and/or panic disorder on the MINI-International Neuropsychiatric Interview [26], co-diagnosed with hypertension, diabetes, and/or ischemic heart disease, and able to consent to participate and be followed for 12 months.

Prospective participants were evaluated for hypertension (elevated systolic blood pressure > 140 mmHg and/or diastolic blood pressure > 90 mmHg), diabetes (capillary blood sugar ≥160 mg/dl), and angina (positive score on Rose Angina Questionnaire) [27] and/or being on treatment for hypertension, diabetes mellitus or ischaemic heart disease. At least one of these diagnoses was required in order to be eligible for enrolment.

Participants unable to provide consent due to cognitive impairment (Modified Short Blessed Cognitive Scale > 7) [28], unable to provide contact information, and those on anti-depressants at the time of initial screening, were excluded from the study. Two recruitment methods were used. Weekly health fairs with free health check-ups were held during the five-week recruitment period. We also screened participants attending PHCs. Nurses and community health workers called ASHAs (Accredited Social Health Activists) conducted the health fairs while ASHAs raised awareness through announcements via community events, posters, and door-to-door visits to community members [29]. Either our assessment staff or ASHAs under supervision of our staff conducted the initial screening. Participants meeting the eligibility criteria of this initial screening were invited to a confirmatory screening at the PHC by the study’s assessment staff. Participants who met the eligibility criteria during the confirmatory screening received a copy of the study information sheet and informed consent. Participants provided written informed consent. Illiterate participants had the option of providing either verbal consent or a thumbprint. In such cases, a witness, unaffiliated with the study, also signed the consent form. The study protocol has been published previously [25].

## Collaborative care intervention

The CCM consisted of two components: a clinic-based intervention (at cluster level) and a community-based intervention, targeting the individual participants, living in the catchment areas of the CCM PHCs. As part of the clinic-based intervention, the participants received additional diagnostic testing and clinical treatment for both their mental illness and chronic disease by the PHC care team that included a physician, a nurse, and a pharmacist. All three were trained in comprehensive integrated mental health and physical health care by study-affiliated psychiatrists and community medicine physicians. They received a full day of training that included didactic lectures, case discussion with role play and question and answer sessions, and their knowledge was assessed before and after the training [30]. During the intervention, the staff in collaborative care PHCs received support via weekly phone calls from consultant psychiatrists based at St John’s Medical College. In addition, adopting the principles of stepped care [31], participants at high risk for suicide as indicated by their responses to the suicidal assessment questions from the MINI [26] were referred to a district hospital psychiatrist for further management, and participants with abnormal laboratory values were referred to relevant specialists as needed.

The community-based intervention comprised of “healthy living” group (HLG) meetings in an accessible community venue, designed to target risk factors important in the management of depression, DM, and CVD. Each group included a maximum of ten same-sex participants attending the same PHC. Groups were formed on an ongoing basis, as enrolment at a given PHC progressed. The first 12 sessions occurred weekly and were facilitated by a master’s level counsellor and co-facilitated by an ASHA. They were followed by nine monthly sessions in which ASHAs were encouraged to take the lead. The behavioural change strategies were based on principles of social cognitive theory such as observational learning, setting manageable goals, practice and getting feedback, building self-efficacy, and skills training. The details of the topics covered have been described in the protocol paper published previously [25]. Briefly, the topics covered were behavioural risk factors linked to CVD and diabetes, strategies to reduce depression, anxiety, and stress and enhance psychological well-being, develop plans to eat healthier food, need for regular physical exercise, strategies for quitting alcohol and tobacco use, identifying successes and barriers to behaviour change and utilizing group support. Participants were strongly encouraged to always attend their originally assigned group’s meetings, but in case of scheduling conflicts, making up a session in another same-gender group’s meeting at the same PHC, was possible.

Finally, ASHAs met with every participant’s family during bi-monthly home visits and encouraged them to support the participant’s new healthy lifestyle.

Implementation and adherence to intervention protocols were documented and monitored through weekly reports of HLG sessions and observation of the sessions by an independent monitor who completed a checklist to ensure that all components were covered, and during weekly consultation calls between PHC physicians and the consulting psychiatrist. All intervention study staff were trained and certified in all components of the intervention and received booster sessions as needed.

## Collaborative care staff training

Staff in the 24 intervention PHCs received training sessions in the CCM by psychiatrists and community medicine physicians from St John’s Medical College. The PHC staff training was designed to facilitate the integration of treatment of depression into their regular practice for patients co-diagnosed with chronic medical conditions. The PHC staff underwent one full day of interactive group training. In the morning all staff of the PHCs was provided training on the management of chronic non-communicable diseases at the clinic level, and the afternoon was devoted to the management of depression using a CCM. Primary care physicians were trained to identify and treat patients presenting with depression and to refer as appropriate. The training of PHC physicians in the use of antidepressant medications was based on the World Health Organization mental health gap guidelines (mhGAP) for treatment of mental disorders in primary care, which recommends treating moderate-to-severe depression with antidepressants [32]. The PHC nurses were trained to function as “care managers” and helped with tracking patient progress and overseeing ASHAs. Support for treatment was provided through weekly calls with consultant psychiatrists who provided consultations for difficult cases and referral recommendations as needed. PHC pharmacists were trained to educate patients and their caregivers regarding medication regimens, side effects, and adherence. The ASHAs were trained in risk factor screening and modification and acted as a liaison between the PHC, patients, and families. They also provided appointment reminders. ASHAs also co-facilitated the healthy living groups.

## Enhanced standard treatment

Standard treatment of depression in the PHC often includes ineffective symptomatic treatment [2]. Hence, for ethical reasons, PHC physicians in the 25 control PHCs received basic training in the treatment of depression per standard treatment protocols from the state’s health department, including appropriate use of antidepressants targeting levels of depression. We also ensured that any patient who was diagnosed as moderately to severely depressed had access to effective treatment through referral to a district hospital psychiatrist. Similarly, patients identified at high risk for suicide as indicated by their responses to the suicidal assessment questions from the MINI [26] during the assessment interviews were referred by the PHC physicians to district hospital psychiatrists for further management. Table 1 provides a summary of both treatment arms.

**Table 1:** Summary of treatment components

	Collaborative care	Enhanced standard care
PHC level:	•Training of the PHC care team in the treatment of mental health and chronic disease according to the collaborative, stepped care model, including antidepressant use	•Basic training of PHC physician in established clinical protocols from the State (Karnataka), including for antidepressant use
	•PHC nurse as case manager	
	•Pharmacist trained in identifying common adverse side effects of antidepressants and educating participants about them	
	•Weekly phone calls between PHC physician and study psychiatrist	
	•Referrals to district psychiatrist for suicidal patients	•Patients with moderate or severe depression or suicidality are referred by study team to district psychiatrist
	•Referrals for abnormal lab values	
Participant level:	•Healthy living groups to develop and maintain skills for improved mental and physical health that can be incorporated into their lifestyle: 12 weekly sessions, followed by 9 monthly sessions	None
	•ASHA support with appointment keeping, getting family support	

*ASHA* Accredited Social Health Activist, *PHC* Primary Health Center

## Randomization and blinding

Randomization was at the PHC rather than the individual level given the involvement of the PHC staff in the clinic-based part of the intervention. Given the PHCs’ small size, a CCM approach required training all their medical staff, and hence it would be impossible to avoid contamination of the control arm if the same staff would subsequently be required to treat some patients according to the CCM, but others in the standard way. Furthermore, participants themselves would find out about the other treatment arm from each other.

Originally 50 of the 61 PHCs in the district were randomly selected and assigned an identification number. Another two PHCs were used to pilot the procedures and measures. All district PHCs were eligible and they were randomized a priori, by the study statistician (EH) using a pseudo-random generator. We had originally intended to also test two recruitment methods: recruitment among patients visiting the PHC only vs. recruitment via specially organized village health fairs. For randomization, the PHCs were first, a priori randomized 1:1 over the two recruitment arms. Subsequently half in each recruitment arm were randomly allocated to one of the treatment arms. Shortly after the start of the study however, due to slow enrolment in the PHC-only method, health fairs were organized in the catchment areas of all PHCs. During the course of the study, six of the PHCs originally randomized to the intervention group were unable to participate, two due to not having a qualified physician on staff, two due to the physician’s unwillingness to participate, one due to a PHC having insufficient patient visits, and one due to a lack of local ASHAs to help implement the community-based intervention. Replacements were drawn from PHCs that were not part of the original random selection or piloting. These replacement PHCs did not know their study arm allocation before joining the study. Ultimately 24 PHCs delivered collaborative care, and 25 enhanced standard care to 2486 participants.

Only study assessment staff members were blinded to treatment arm allocation.

## Assessment procedures

Baseline data were collected between May 2015 and November 2018. The 12-month follow-up was completed in November 2019.

All study measures have been used previously in India. They were translated into Kannada and back-translated. Cohort participants were assessed at baseline, three months, six months, and 12 months. Trained interviewers administered face-to-face interviews to the participants at the PHCs or another mutually convenient location that offered privacy. To minimize attrition, we collected extensive contact information from all participants, including mobile phone numbers as well as street addresses, landmarks, and the name and phone number of someone who always knew how to reach the participants. All research materials were coded with ID numbers only and linked to contact information on a separately stored document kept under lock and key.

## Outcomes

Outcomes pertained to the individual participant level. We used Kessler-10, a brief standardized questionnaire that correlates well with other commonly used depression screening questionnaires to do the initial screening for psychological distress [33, 34]. At the subsequent confirmatory screening, MINI [26, 35] was used to confirm the diagnosis of major depressive disorder, dysthymia, generalized anxiety disorder, and/or panic disorder as per DSM-IV guidelines. During the baseline and follow-up assessments of the trial, the primary outcome severity of depression was assessed with the Patient Health Questionnaire Depression Scale (PHQ-9) [36, 37]. The PHQ-9 is a 9-item self-report measure of depressive symptoms in the past 2 weeks with a total score ranging from 0 to 27 and higher scores indicating greater severity of symptoms.

## Sample size

Sample size estimates and power calculations have been described previously [25]. The estimated sample size of 1250 in each intervention arm (50 participants per PHC; 50 PHCs) was based on an attrition rate of 20%, and an intra-class correlation (ICC) of 0.1 to account for clustering of participants in PHCs. Pooling data across the three post-intervention measurements per person and adjusting for repeated measures with an assumed ICC = 0.5, results in a final effective sample size of *n* = 306 person-time observations per group. This allows 80% power to detect the expected effect size based on previous results [12, 38, 39], i.e. a difference of 40% vs. 52% of control vs. intervention arm participants, respectively, recovering to below threshold levels for depression and CVD/DM risk (with α = 0.05/2 = 0.025). The current analyses focus on the mental health outcome; the physical health results comprise several measures and will be reported separately.

## Statistical analyses

The baseline sample was described via frequencies and percentages for categorical variables, and mean plus standard deviation (SD) for continuous variables.

The effect of the intervention was assessed via intention-to-treat (ITT) analyses, though the ITT principle was slightly modified by the fact the replaced PHCs were all in the intervention arm, as outlined above. We compared mean PHQ-9 scores at each wave between the two treatment groups via a univariable linear regression model with standard errors adjusted for clustering of participants in PHCs by using the clustered sandwich estimator of variance. We also ran a multilevel linear regression model of the continuous PHQ-9 outcome with waves nested within participants and participants nested within PHCs. A multi-level model adequately accounts for the dependence among the observations due to (1) the repeated measurements and (2) participants attending the same PHC and makes optimal use of all available data [40, 41]. Wave (baseline and all three follow ups, treated categorically) and treatment arm were included as fixed covariates, as was the interaction between these two variables, to test if the change in the depression scores from baseline to follow-up was significantly different in the enhanced standard care vs. the collaborative care arm. We specified random intercepts for participant (level 2) and PHC (level 3). Though treatment randomization was stratified by recruitment condition, we did not retain recruitment arm as a covariate as is normally recommended for randomization stratification variables [42], because recruitment was switched to include health fairs everywhere (see above), and analyses showed the variable had indeed no effect (see Results in Supplement). Analyses were performed in Stata 17.0.

## Role of the funding source

The study funders had no role in study design, data collection, data analysis, interpretation of the data, or writing of the manuscript. KS, EH, and ME had full access to all study data. All authors had final responsibility for the decision to submit for publication.

## Results

**Fig. 1:**
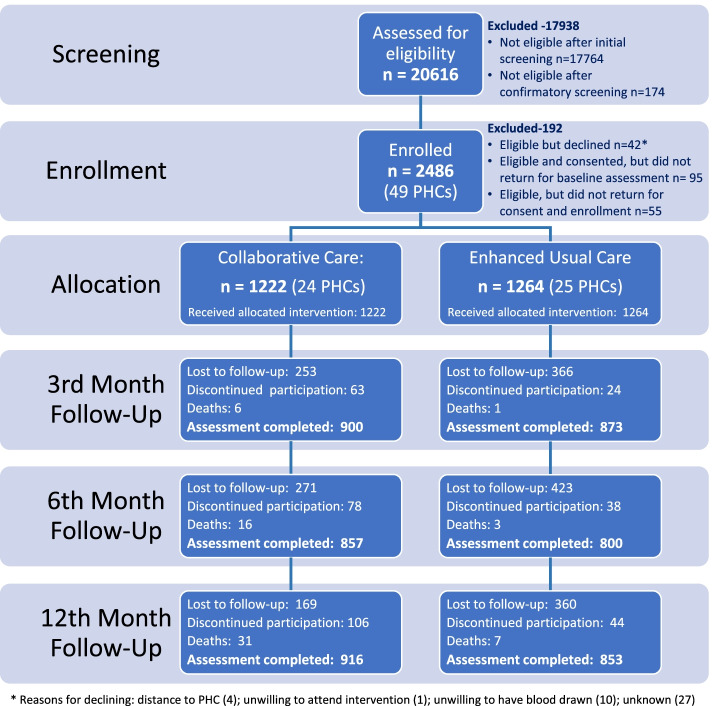
Participation details of the trial

Figure 1 shows the flow of participants throughout the trial. A total of 2486 participants were enrolled at baseline; 1222, from 24 PHCs, in the collaborative care arm, and 1264, from 25 PHCs, in the enhanced standard care arm. A description of the baseline sample is presented in Table 2.

**Table 2:** Socio-demographic characteristics of the sample at baseline, by treatment arm (*n* = 2486)

	Collaborative care (***n*** = 1222; 24 PHCs)		Enhanced standard care (***n*** = 1264; 25 PHCs)		All (***n*** = 2486)	
	n	%	n	%	n	%
Female	947	77.5	917	72.5	1864	75.0
Marital status:						
Married	754	61.7	837	66.2	1591	64.0
Widowed	443	36.3	400	31.6	843	33.9
Other	25	2.0	27	2.1	52	2.1
Education^a^						
No formal education	738	60.5	700	55.4	1438	57.9
Primary education	352	28.9	374	29.6	726	29.2
≥ Secondary education	130	10.7	189	15.0	319	12.8
Monthly household income in Rupees:						
≤ 5000	877	71.8	893	70.6	1770	71.2
5001–10,000	263	21.5	300	23.7	563	22.6
> 10,000	82	6.7	71	5.6	153	6.2
Hindu religion	1207	98.8	1241	98.2	2448	98.5
Age categories (in years):						
30–44	88	7.2	91	7.2	179	7.2
45–54	237	19.4	261	20.6	498	20.0
55–64	440	36.0	436	34.5	876	35.2
65–74	382	31.3	399	31.6	781	31.4
≥ 75	75	6.1	77	6.1	152	6.1
Number of chronic conditions						
1	332	27.2	338	26.7	670	27.0
2	372	30.4	412	32.6	784	31.5
3	461	37.7	458	36.2	919	37.0
4	57	4.7	56	4.4	113	4.5

^a^ missing data for 3 subjects

Most participants were female (75.0%) and married (64.0%); a third were widowed (33.9%). Over half reported no formal education (57.9%) and a monthly household income of less than 5000 Indian Rupees (71.2%), well below the per capita monthly income of INR 8930 for the period between 2016 and 2017 [43]. Two thirds of participants were between 55 and 74 years old; mean (SD) age was 59.2 (10.0) years. The sample was almost exclusively Hindu (98.5%). The majority of participants (73.0%) had more than one co-morbid medical condition, calculated as the sum of a participant’s positive diagnoses for diabetes, hypertension, hyperlipidemia, and angina.

At baseline, the mean observed PHQ-9 depression score was about 8.5 in both treatment conditions (Table 3). As current anti-depressant use was an exclusion criterion, only one person was on antidepressants at baseline, having started them after immediate referral during screening, as per protocol. Subsequently, anti-depressant use was reported by around a quarter of collaborative care participants in each of the follow-up waves, vs. only around 1% in the enhanced standard care arm. A total of 365 participants (29.9%) in collaborative care vs. 22 (1.7%) in enhanced standard care reported anti-depressant use at any point in the study.

**Table 3:** Depression scores (PHQ-9) and anti-depressant use by wave and treatment arm

		Treatment arm				Difference in change from BL between arms	
Wave	Variable	Collaborative care (24 PHCs)		Enhanced standard care (25 PHCs)			***p***-value
Baseline	On antidepressants: n (%)	1	(0.1%)	0	(0%)		
	PHQ-9 score: observed mean (SD)	8.47	(3.95)	8.58	(4.29)		
3 months	On antidepressants: n (%)	233	(25.9%)	8	(0.9%)		
	PHQ-9 score: observed mean (SD)	5.24	(3.21)	6.69	(3.73)		0.002^a^
	Predicted change in PHQ-9 from BL^b^	−3.11		−1.83		−1.28	< 0.001
6 months	On antidepressants: n (%)	223	(26.0%)	10	(1.3%)		
	PHQ-9 score: observed mean (SD)	4.81	(2.98)	6.13	(3.42)		< 0.001^a^
	Predicted change in PHQ-9 from BL^b^	−3.52		−2.22		−1.31	< 0.001
12 months	On antidepressants: n (%)	252	(27.5%)	10	(1.2%)		
	PHQ-9 score: observed mean (SD)	4.22	(2.65)	5.23	(2.71)		< 0.001^a^
	Predicted change in PHQ-9 from BL^b^	−4.06		−3.00		−1.06	< 0.001

^a^ Based on univariable regression of PHQ-9 score on treatment arm per wave, using cluster (PHC) robust standard errors

^b^ Based on multi-level linear regression of the repeated measurements of PHQ-9 on wave, treatment arm and wave*treatment arm, with random intercepts for participant and PHC (ICC_PHC_ = 0.13, ICC_Partic.|PHC_ = 0.31)

**Fig. 2:**
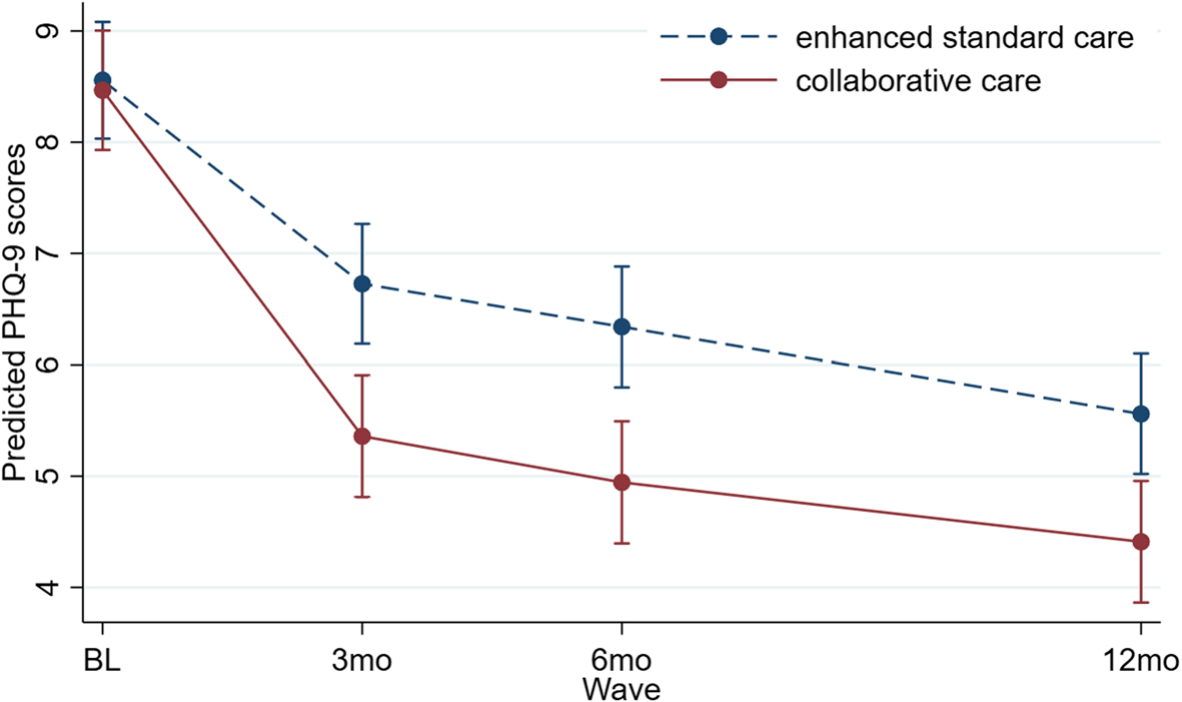
PHQ-9 depression scores over time by treatment arm, with 95% CI

Mean depression scores had decreased in both treatment arms by the three months follow-up but the drop was significantly larger in the collaborative care arm (mean PHQ-9 = 5.24; SD = 3.21) than in the enhanced standard care arm (mean PHQ-9 = 6.69; SD = 3.73; *p* = 0.002). Depression scores decreased further slightly in subsequent waves, remaining significantly lower in the treatment arm than the control arm (see Table 3 for details). The mixed-effects longitudinal analysis (Fig. 2 and Table 3) confirmed the abovementioned results observed at the individual follow-up waves. The joint test for the interaction effect between treatment arm and wave was statistically significant (overall χ^2^ (3 df) = 71.52, *p* < 0.001), as were the tests for the three individual coefficients making up the interaction; the decrease from baseline in mean depression scores was significantly larger in the collaborative care arm than the enhanced standard care arm at each follow-up. Figure 2 shows the predicted PHQ-9 scores at each wave by treatment arm.

## Discussion

To our knowledge, this is the first cluster randomized controlled trial of the Collaborative Care Model for patients with depression and co-morbid medical conditions from rural India. Our study showed that recipients of collaborative care reported significantly reduced depressive symptoms compared to the control group starting at three months follow up, and the difference was maintained until the study’s endpoint, 12 months after the start of the trial. Not surprisingly, given that this was a focus of the training and weekly consults, the proportion of participants that reported using antidepressant medications at any time during the study was much higher among participants attending intervention PHCs compared to those attending PHCs assigned to the enhanced standard care arm.

Several systematic reviews have demonstrated that CCM is associated with significant improvement in depression outcomes compared to usual care [22, 23, 44]. However, studies examining the effectiveness of CCM on depression outcomes in LMIC settings, particularly among rural population are limited. The few studies that did examine the effects of collaborative care on depression outcomes in LMIC primary care settings did not specifically include co-morbid medical conditions [24, 38]. Thus, our study fills a critical gap in the literature on the effectiveness of collaborative care on depression outcomes with well-defined co-morbid medical conditions among rural participants in LMIC settings. The modest difference in mean depression scores on PHQ-9 at six months between the control and intervention group (6.13 vs. 4.81) is in agreement with findings from earlier studies on depression outcomes in participants with co-morbid medical conditions [45]. However, it is important to note that at six months, the mean PHQ-9 score among participants in the intervention PHCs had dropped to below five, putting them in the ‘no depression’ range. The difference in depression outcomes between the two arms in the present study could have been attenuated as physicians in the control PHCs received some training too, in the state standards for the management of depression, which could have influenced their interactions with participants. The majority of participants in the present study had PHQ 9 scores that were in the mild-to-moderate category of severity of depression at baseline. Participants with dysthymic disorder were also included, which aligns with recent studies that recommend inclusion of patients not only with a diagnosis of major depression but also dysthymia while screening for depressive symptoms in patients with co-morbid medical conditions [46, 47]. Studies have shown that both dysthymia and sub-threshold depressive symptoms are risk factors for recurrences of subsequent episodes of major depression in patients with chronic medical conditions [48].

The findings from our study also provide support for integrating treatment for depression and chronic medical conditions [14, 15]. CCM is a complex set of interventions that includes a trained primary care physician, care manager, and a consulting psychiatrist [49]. One of the novel features of our CCM was the inclusion of ASHAs. In India, shifting to lower-level health providers such as ASHAs for the ongoing support of health delivery services has primarily occurred in the field of maternal and child health and less in the area of chronic medical conditions including mental health. In the present study, ASHAs were trained in risk factor screening and modification, acted as a liaison between the participating PHCs, patients, and families, and co-facilitated HLG sessions, thereby ensuring greater engagement among participants with the intervention [29]. While ASHAs have been used in the past to improve access to mental health care [50], to our knowledge, ours is the first study where trained ASHAs were part of a collaborative care team. In LMIC, access to mental health services and adherence to treatment recommendations remains a challenge and ASHAs can play a critical role in linking rural patients to PHCs, reducing loss to follow-up and thus ensuring continuity of care.

There was a higher use of antidepressant medications among the participants in the intervention group compared to the control arm. The use of antidepressant medications for the treatment of moderate-to-severe depression is a critical component of CCM [51]. Studies from India have noted that adherence to antidepressant medication is poor among patients attending PHCs [24]. A recent cluster RCT from India showed that antidepressant medication adherence was better among participants attending intervention PHCs compared to the usual care PHCs [52]. A systematic review of CCM on depression outcomes reported higher antidepressant medication use and better adherence among participants with co-morbid medical conditions in the intervention arm [23]. The same group of authors posit that perhaps participants with co-morbid medical conditions are more responsive to a structured management program offered by CCM as they are already used to taking medication for management of chronic physical conditions. In addition, the systematic review concluded that studies that relied on systematic identification of participants through the use of screening questionnaires and diagnostic interviews to diagnose depression as compared to recruitment by clinicians predicted increased use of antidepressant medications [23]. Findings from our study of increased antidepressant use among participants in the intervention arm aligns with this observation and highlight the importance of population-based strategies and the use of a structured approach to diagnosis and care of depression.

Ours is one of the largest studies that have examined the effectiveness of CCM on depression outcomes in rural participants with co-morbid medical conditions in an LMIC setting. Screening, diagnosis, and severity of depression were measured using well-validated questionnaires. We also assessed fidelity to various components of treatment intervention.

In addition to these strengths, the study also has some limitations, including an inability to generalize beyond the geographical region targeted. The need to replace six PHCs in the intervention arm, implies that the replacement PHCs, though randomly selected from the remaining available PHCs, could have introduced bias. Four of these six had to be replaced due the PHC physician’s being unqualified or unwilling to deliver the intervention, reasons that cannot apply to the control arm PHCs, as they were simply delivering standard care. Finally, the main outcome measure was based on a self-report. Repeated administration of the self-report measure could thus have resulted in socially desirable response bias in both study arms. However, a recent meta analysis concluded that the results of CCM effects on depression outcomes across studies were similar regardless of whether the outcome measure was based on the self-report or an objective measure of depression, thus reducing the likelihood of such bias [45].

In conclusion, our finding that implementation of a CCM improves depression outcomes among rural participants with mild-to-moderate depression and co-morbid medical conditions has potential implications for integrating mental health and chronic disease treatment. Through task shifting with the involvement of non-mental health workers, there is a potential for scaling up the CCM intervention across PHCs as part of India’s National Health Mission. Future studies should examine the impact of depression outcomes on physical health outcomes particularly among those with multiple chronic medical conditions.

## Supplementary Information

**Additional file 1.** Supplemental results recruitment arm covariate.docx: Fixed effects estimates for regression of PHQ-9 depression score on wave and treatment arm, with recruitment arm as covariate.

## Data Availability

The datasets used and/or analysed during the current study are available from the corresponding author on reasonable request.

## Abbreviations

ASHA: Accredited Social Health Activist
CCM: Collaborative Care Model
CVD: Cardiovascular Disease
DM: Diabetes Mellitus
HLG: Healthy Living Group
ICC: Intra Class Correlation
ITT: Intention to Treat
LMIC: Low and Middle Income Countries
PHC: Primary Health Center
RCT: Randomized Controlled Trial
SD: Standard Deviation
