# Supplementary material for: Collaborative care compared to enhanced standard treatment of depression with co-morbid medical conditions among patients from rural South India: a cluster randomized controlled trial (HOPE Study)
Source: BMC Psychiatry. 2022 Jun 13;22:394. doi: 10.1186/s12888-022-04000-3 (PMC9195442; doi:10.1186/s12888-022-04000-3)
Supplement: Supplementary file 1 — Additional file 1. Supplemental results recruitment arm covariate.docx: Fixed effects estimates for regression of PHQ-9 depression score on wave and treatment arm, with recruitment arm as covariate. [file 12888_2022_4000_MOESM1_ESM.docx]

| **Fixed effects estimates for regression of PHQ-9 depression score on wave and treatment arm, with recruitment arm as covariate** | | | | |  |
| --- | --- | --- | --- | --- | --- |
|  | **Coefficient** | **(95% conf. interval)** | | **p-value** | |
| Wave (ref. = baseline) |  |  |  |  | |
| 3 months | -1.83 | (-2.09; | -1.57) | <0.001 | |
| 6 months | -2.22 | (-2.48; | -1.95) | <0.001 | |
| 12 months | -3.00 | (-3.26; | -2.74) | <0.001 | |
| Collaborative care treatment | -0.09 | (-0.84; | 0.65) | 0.815 | |
| Wave by treatment interaction |  |  |  | <0.001 | |
| 3mo. * collab. care | -1.28 | (-1.64; | -0.92) | <0.001 | |
| 6mo. * collab. care | -1.31 | (-1.68; | -0.94) | <0.001 | |
| 12mo * collab. care | -1.06 | (-1.42; | -0.70) | <0.001 | |
| Enhanced recruitment arm | 0.27 | (-0.46; | 0.99) | 0.468 | |
| (Intercept) | 8.43 | (7.80; | 9.06) | <0.001 | |
| Note: model included random intercepts for Participant and PHC to account for nested data structure | | | | |  |
